# Actinomyces radingae Infection at an Unusual Location: A Case Report

**DOI:** 10.7759/cureus.108449

**Published:** 2026-05-07

**Authors:** Steven Sochacki

**Affiliations:** 1 Family Medicine, Atrium Health Floyd, Rome, USA

**Keywords:** actinomyces, atypical infection, augmentin, bactrim, dermatology, diabetes mellitus type 2, radingae, skin lesion, unusual sites

## Abstract

The Actinomyces genus of bacteria rarely causes infections in humans, as its members are part of the oral or skin flora. However, they have been known to cause infections when a person is immunocompromised. This case report describes a 65-year-old patient with type 2 diabetes mellitus infected with *Actinomyces radingae* on the back of his neck, with no known inciting incident other than having a neck lesion in that location, which has been stable for the past 35 years. This area was cultured, and the organism isolated was *A. radingae*. Antibiotic treatment was initiated, and his follow-up appointment was scheduled toward the end of the treatment course. The infection appeared to have subsided, and he was referred to dermatology, as the lesion on his neck that had previously been stable for the past 35 years still persisted. In conclusion, even rare Actinomyces species with unusual presentations can usually be treated with standard antibiotics in an outpatient setting.

## Introduction

The Actinomyces genus encompasses a wide variety of Gram-positive, facultative anaerobic bacteria that can be found in soil and water [1]. These thin, branching bacilli can also be found on mucosal surfaces as normal inhabitants of the human mouth, gastrointestinal (GI), and genitourinary (GU) tracts [2]. They are well known to cause infections involving the joints, skin, soft tissue, and mouth [3]. The most common site of infection, accounting for about 60% of cases, is the cervicofacial area [2]. One of the most common pathogenic species in humans is Actinomyces israelii, but there are several other species that can cause pathology [3]. Although infections due to Actinomyces may result in pain, fever, or abscess formation, most infections do not appear to present with notable clinical features [3]. There is evidence that these species tend to infect people who are immunocompromised, including but not limited to patients with alcohol use disorder, tobacco use disorder, poor dentition, and diabetes [1]. Infections due to Actinomyces species require antibacterial therapy, which may include penicillins, macrolides, or lincosamides [1]. Metronidazole is not recommended, as multiple species are resistant [4]. The duration of therapy is based on the severity of the infection, the type and route of antibacterial agent used, and the location of the infection [1]. Surgery can also be used in the treatment of severe actinomycosis along with IV antibiotic therapy, which may last for months [5].

## Case presentation

**Figure 1:**
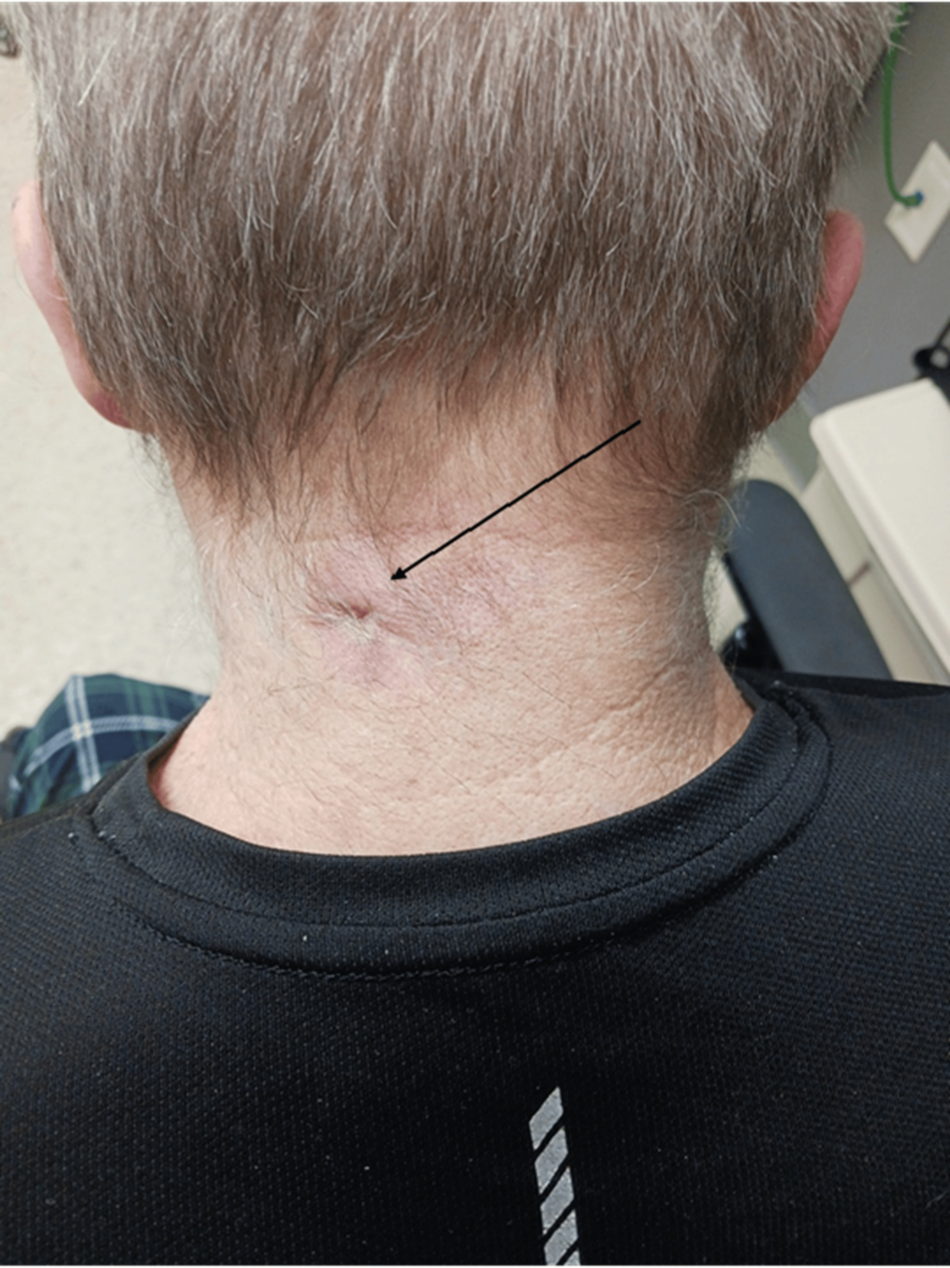
First follow-up visit Erythematous neck lesion with an entrance to the pouch. The patient gave verbal permission for the image to be used.

The patient who is the subject of this report is a 65-year-old male, a current cigarette smoker with type 2 diabetes, who presented to our family medicine clinic in northwest Georgia with complaints of white, purulent drainage and non-radiating pain involving a lesion on the back of his neck. The patient had a neck lesion, specifically a pouch on his neck, which started about 35 years ago and suddenly began enlarging in size three weeks prior to presentation (Figure 1). He denied any other symptoms related to the neck lesion except for drainage and pain. On physical examination, he had constant 5/10 posterior neck pain, and a 5 cm erythematous lesion was present near the midline of his posterior neck. He was afebrile, and his other vital signs were within an acceptable range. Labs were not obtained for this patient due to cost and a presentation similar to that of routine skin abscesses. A culture was then taken from the drainage of the ruptured lesion. He was initially started on trimethoprim-sulfamethoxazole 800 mg/160 mg (TMP-SMX) twice a day for seven days, as this is a typical treatment for a skin abscess. He presented for re-evaluation a couple of days later and reported no side effects from TMP-SMX. On examination, there did not appear to be any significant change from the initial encounter.

**Figure 2:**
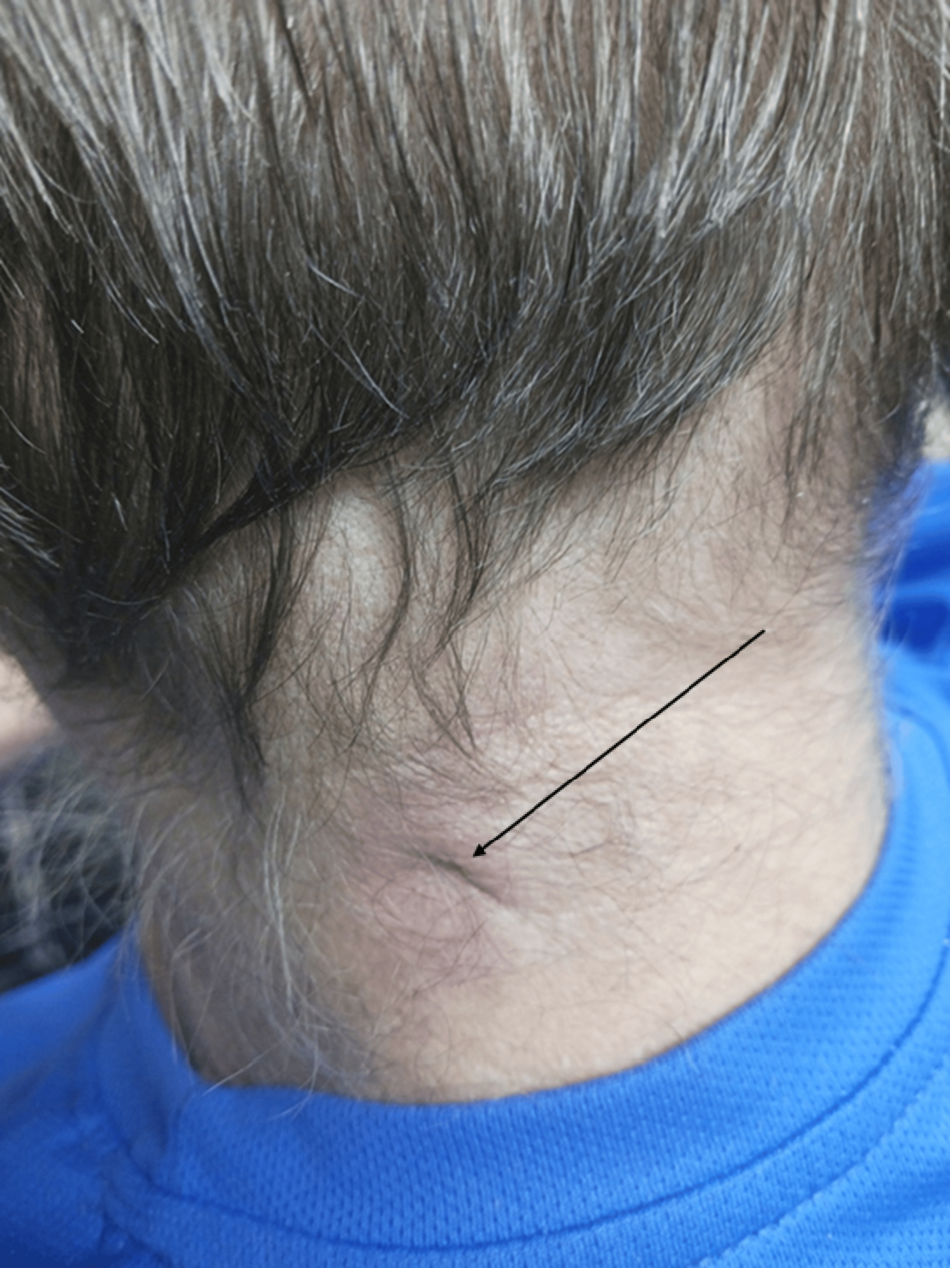
Near the end of the treatment course at the second follow-up visit Slightly less erythematous lesion compared to the previous visit after about two weeks of antibiotic treatment. The patient gave verbal permission for the image to be used.

A few days later, the Gram stain showed no organisms. However, the aerobic culture grew *Actinomyces radingae*, so the medication he had been prescribed was switched to amoxicillin/clavulanic acid 875 mg/125 mg to be taken twice a day for two weeks. At the final visit, after the course of amoxicillin/clavulanic acid had nearly been completed, he reported less pain in the region and notably decreased drainage compared to before. The erythema noted on physical examination had also decreased compared to the previous visit (Figure 2).

He was then referred to dermatology for definitive treatment due to the chance of reinfection, as the lesion on his neck that was previously stable was still present. He also gave verbal consent for his case to be used in a publication.

## Discussion

Actinomyces species are relatively uncommon causes of infection, and *A. radingae* is very rare among them. Only a handful of case reports have been published involving this species, and none have involved the posterior portion of the neck [2]. The typical Actinomyces infection involves a slowly growing, painless mass in the cervicofacial area, mostly in the tissues of the mandible. These masses can become abscesses that express yellow-colored sulfur granules [3]. In our case, there were no sulfur granules, and the patient reported pain with regard to the lesion. The infection was subacute, making it similar to other Actinomyces infections in that aspect. The patient did have type 2 diabetes, which likely predisposed him to this infection [3,4]. Immunosuppression, as a result of HIV, malignancy, or immunosuppressant medications, for example, does seem to make infection with Actinomyces more likely and allow it to develop in more unusual locations like the leg, the abdomen, or diffusely [6-8]. Once the Actinomyces species was isolated, the medication was switched to Augmentin due to notable sensitivity to beta-lactams [4,9]. However, Bactrim may have been able to treat the infection as well [3]. The choice to switch to Augmentin was also made because Augmentin has fewer serious side effects compared to Bactrim [10,11].

There were a few limitations involving this case. One is that only a single patient was treated, so it is unclear how the treatment would apply to other patients with a similar infection. Another is that the patient already had a lesion that had been present for years, which most patients would not have. An extensive workup with regard to labs and imaging was not done, as the cost to the patient was a limiting factor. At last, no susceptibility testing was available per the lab to which the culture was sent.

## Conclusions

*A. radingae* is a rare cause of skin infection that would be unlikely to be seen in a clinical setting. In our case, it did not have any notable features compared to other abscesses. However, the treatment was similar to what would be done if the patient were infected with other Actinomyces species. In conclusion, *A. radingae* is one of many rare Actinomyces species that could appear in a wound culture, and more research should be done to further understand how it can present in a patient and the optimal treatment once identified.
